# Bacterial agents of the discharging middle ear among children seen at the University of Nigeria Teaching Hospital, Enugu

**DOI:** 10.11604/pamj.2017.26.87.9243

**Published:** 2017-02-21

**Authors:** Gideon Chukwudalu Ilechukwu, Chioma Azuka Ilechukwu, Agozie Chukwunedum Ubesie, Ijeoma Okoroafor, Basil Chukwuemeka Ezeanolue, Ngozi Chinyelu Ojinnaka

**Affiliations:** 1 Department of Paediatrics, Whiston Hospital, Prescot, United Kingdom; 2 Department of Paediatrics, University of Nigeria, University of Nigeria Teaching Hospital, Enugu; 3 Department of Otorhinolaryngology, University of Nigeria/University of Nigeria Teaching Hospital, Enugu

**Keywords:** Bacterial agents, discharging middle ear, Nigerian children

## Abstract

**Introduction:**

Discharging middle ear continues to be one of the commonest problems seen in the developing world. There is an ever growing need to carry out studies periodically to determine the common bacterial agents responsible for discharging otitis media and their antibiotic sensitivity especially in set-ups characterized with minimal laboratory services. The study sought to determine the common bacterial agents causing discharging middle ear among children presenting at the University of Nigeria Teaching Hospital, Enugu and their sensitivity to the commonly available antibiotics.

**Methods:**

Middle ear swabs were collected from 100 children aged 1 month to 17 years at the Children Out-Patient and Otorhinolaryngology Clinics of the University of Nigeria Teaching Hospital, Enugu, Nigeria. The specimens were cultured for aerobic bacterial organisms and their sensitivity determined.

**Results:**

Among those with acute discharge, *Staphylococcal aureus* was isolated in 31.3% and Proteus species in 25.0%. In chronically discharging ears, Proteus Species dominated (39.1%), followed by *Staphylococcal aureus* (28.3%).

**Conclusion:**

*Staphylococcal aureus* and Proteus species were the commonest bacterial agents in acute and chronic otitis media respectively. Most isolates showed high sensitivity to the fluoroquinolone antibiotics.

## Introduction

Otitis media is one of the most common infectious diseases of childhood worldwide [1]. It is the inflammation of the mucous membrane of the middle ear cleft which includes the middle ear cavity, mastoid antrum, the mastoid air cells and the Eustachian tube [2]. When the inflammation is associated with a discharge from the ear through a perforation in the tympanic membrane or via a ventilatinig tube, suppurative (or discharging) otitis media results. Otitis media may be acute (less than 6 weeks) or chronic (at least 6 weeks) [1]. Discharging ear or otorrhea is drainage exiting the ear which may be serous, serosanguineous, or purulent [3]. Bacteria have remained the most important aetiological agents in suppurative or discharging otitis media. While non-typable *Haemophilus influenzae, Streptooccus pneumonia* and *Moraxella catarrhalis* are commonly reported as aetiological agents in acutely discharging ears in the developed countries [4], local studies done in Nigeria suggest that *Moraxella catarrhalis* is not a predominant organism in acutely discharging otitis media in Nigerian children [5]. *Pseudomonas aeruginosa* is commonly implicated in chronic discharging otitis media in Nigerian children [5, 6]. Complications of discharging otitis media are numerous and include hearing impairment, mastoiditis, facial nerve paralysis, cholesteatoma, tympanosclerosis, bacterial meningitis and brain abscess, to mention a few. Treatment of otitis media is usually based on empiric knowledge of aetiologic organisms and their sensitivity pattern. There is emerging evidence of multi-drug resistance of bacterial isolates with reduction in antibiotic efficacy [7, 8]. Many paediatricians and general practitioners base their treatment of otitis media on empiric evidence of the aetiologic agents and their sensitivities to various antimicrobial agents. In view of these, it is important to document the trend in Enugu, to aid in appropriate treatment of this condition and help prevent its complications which may arise if otitis media is not treated or is improperly treated. This paper therefore, aims to present the aerobic bacteriological agents (and their antibiotic susceptibilities) implicated in discharging ears of children in Enugu.

## Methods

***Study site:*** Study was conducted at the Children Out-Patient and Otorhinolaryngology Clinics of the University of Nigeria Teaching Hospital, Enugu, Nigeria.

***Sampling method:*** The convenience sampling technique was used: each consecutive patient seen at the Paediatric and Otorhinolaryngology clinics with ear discharge with or without other symptoms of otitis media was recruited. Data were collected between July and September 2007.

***Sample size calculation:*** The sample size was calculated using the formula: N= Z2 p(100-p)/D2. Where N = minimum sample size, Z = confidence interval (1.96), P = prevalence with reference to a previous study (6%), D = standard error (5%). Substitutions in the above formula give a minimum sample size of 87 participants. Adding an attrition rate of 10% will bring the minimum sample size to 96 participants, rounded up to 100 participants

***Subject recruitment:*** The participants were 100 children aged 0 to 17 years who presented who presented with discharging middle ear. Children with foreign bodies in or infection of the external auditory meatus were excluded; as well as those who had taken antibiotics within the preceding two weeks to their presentation.

***Data collection:*** A structured questionnaire designed for this study was used to record information on participants by two of the researchers. A pilot study to test information collection tool was conducted on 35 patients who qualified for the inclusion criteria. Information collected included the child's name, age, sex, place of abode, parents' educational level and occupation, presenting symptoms of the patient, duration of ear discharge and immunization status of the patient. After otoscopic examination to document presence of perforated tympanic membrane and pus in the middle ear, the external ears were cleaned with normal saline and 70% alcohol solution and allowed to dry for 1-2 minutes. After this, an ear swab from the discharging ear was taken with the aid of sterile cotton-tipped applicators taking care not to touch the skin of the external auditory meatus to limit contamination of the specimen. In patients with bilateral discharging otitis media, samples were collected from only one ear which was preferred by the patient or mother. The swabs were then quickly transported to the microbiology laboratory of the Eastern Nigeria Medical Center Enugu, a nearby specialist center located very close to the University of Nigeria Teaching Hospital Enugu.

***Sample processing and analysis:*** Samples of the ear discharge were promptly plated onto freshly prepared chocolate, blood and cystine-lactose-electrolyte-deficient (CLED) agar. The blood and cystine-lactose-electrolyte-deficient (CLED) agar plates were incubated aerobically at 37°C for 24 hours while the chocolate agar plates were incubated microaerobically using the candle jar extinction technique [9]. After the 24 hour incubation period, the plates were examined for growth and individual colonies were further analysed for their physical characteristics such as morphology, colour (pigmentation), odour and in case of blood agar, haemolysis. No anaerobic cultures were carried out and mycobacteria were not searched for because of unavailability of the required laboratory materials. A Gram stain was carried out on all cultured isolates. Thereafter, characterization to species level was carried out using standard bacteriological methods [10]. Antibiotic susceptibility tests with multi-discs were carried out for various drugs using the disc diffusion method [10]. Choice of the antibiotics to be tested for was based primarily on knowledge of commonly available antibiotic discs. Zones of inhibition for each antibiotic, if formed were then measured to the nearest millimeter and documented. Interpretation of these results in terms of resistance or susceptibility was according to accepted protocol [10].

***Ethical approval:*** Ethical Clearance for this study was obtained from the ethical committee of the University of Nigeria Teaching Hospital Enugu before the study was commenced. Informed written consent was obtained from parents and guardian of the participants and assent was obtained from children where appropriate.

***Data analysis:*** Data were entered into a computer database and were analyzed using SPSS version 11.0. Chi-square tests (χ^2^) were used to test for significance; and probability value (p-value) of less than 0.05 was taken as being statistically significant.

## Results

The subject were aged 1 month to 17years (median 3 years). The male: female ratio was 1:0.9. Majority of the participants (70%) were aged 1month to 5years while 30 (30%) were between 6 and 17years. Fifteen (15.0%) participants were from the upper socio-economic class, while 32 (32.0%) and 53 (53.0%) participants were from the middle and lower socio-economic class respectively. Ninety-one (91.0%) ear swab samples grew isolates on culture while 9(9.0%) were sterile. Out of these 91 samples with positive bacterial yield, 3(3.3%) samples yielded multiple isolates while the rest 88(96.7%) yielded single isolates on culture. On the whole, 94 isolates were got from the bacterial cultures. Forty (42.6%) were gram positive; whereas 54(57.4%) were gram negative. Among the isolates from patients with acute discharging otitis media, gram positives constituted 43.8% while gram negatives formed 56.3% of them. The leading organisms in acutely discharging ears were: *staphylococcus aureus*15(31.3%) followed by Proteus species 12(25.0%), and then by *Pseudomonas aeruginosa*11(22.9%) (Table 1).

**Table 1:** Frequency distribution of cultured isolates in participants with acute and chronic discharging otitis media and their p-values

Isolate	Acute	Chronic	p-value
	Frequency (%)	Frequency (%)	
*Staphylococcus aureus*	15 (31.3)	13 (28.3)	0.92
Proteus species	12 (25.0)	18 (39.1)	0.22
*Pseudomonas aeruginosa*	11 (22.9)	6 (13.0)	0.33
*Streptococcus pneumonia*	3 (6.3)	5 (10.9)	0.67
Klebsiella species	2 (4.2)	3 (6.5)	0.96
*Streptococcus pyogenes*	2 (4.2)	1 (1.6)	
Non haemolytic Streptococcus	1 (2.1)	1 (2.2)	0.99
*Escherichia coli*	1 (2.1)	0 (0.0)	0.99
Neisseria species	1 (2.1)	0 (0.0)	0.99
Total	48 (100.0)	46 (100.0)	

Among the 49 cases of chronically discharging otitis media, gram positive and gram negative organisms accounted for 41.3% and 58.7% of them respectively. The commonest causative isolated agent in these chronic cases was Proteus species 18 (39.1%), followed by *Staphylococcus aureus*13(28.3%). Others were *Pseudomonas aeruginosa*6(13.0%), *Streptococcus pneumoniae*5(10.9%), Klebsiella species 3(6.5%) and Non haemolytic Streptococcus 1(2.2%) (Table 1). No significant difference was found among the isolates cultured in acute and chronic discharging ears (Table 1). As otitis media in children is documented to be commonest in children less than 5 years [8], the pattern of bacterial isolates in those less than 5 years of age and in those at least 5 years of age was sought as is depicted in Table 2. No relationship of statistical significance was observed when chi-square (with Yates correction for continuity) was calculated for the different organisms. *Staphylococcus aureus*and *Streptococcus pneumoniae* each showed 95.7% and 100% sensitivity respectively to ofloxacin; and 75.0% and 100.0% sensitivity respectively to gentamycin. Cloxacillin, and amoxicillin-clavulanate combination were also seen to show good in vitro activity against *Staphylococcus aureus*. However, *Staphylococcus aureus*and *Streptococcus pneumonia* were poorly sensitive to some other commonly used antibiotics like cefuroxime, co-trimoxazole and amoxicillin (Table 3). Proteus species and *Pseudomonas aeruginosa* showed 95.5% and 100.0% sensitivity respectively to ofloxacin; and on the other extreme, they were both 100.0% resistant to cloxacillin. However, gentamycin and to a lesser extent, chloramphenicol had good antibacterial activity against them. While Proteus was 100.0% sensitive to ceftriaxone, *Pseudomonas aeruginosa*was only 66.7% sensitive to it (Table 3). In general, all the major isolates showed excellent *in vitro*sensitivity to ofloxacin, and ciprofloxacin.

**Table 2:** Pattern of bacterial isolates in different age groups and their probability values

Organism	< 5 years	≥ 5 years	p-value
	Frequency (%)	Frequency %)	
*Staphylococcus aureus*	19 (31.7)	9 (26.5)	0.62
*Streptococcus pneumoniae*	5 (8.3)	3 (8.8)	0.99
*Streptococcus pyogenes*	2 (3.3)	0 (0.0)	0.74
Non haemolytic Streptococcus	0 (0.0)	2 (5.9)	0.25
Proteus species	20 (33.3)	10 (29.4)	0.72
Klebsiella species	2 (3.3)	3 (8.8)	0.50
*Pseudomonas aeruginosa*	10 (16.7)	7 (20.6)	0.63
*Escherichia coli*	1 (1.7)	0 (0.0)	0.99
Neisseria	1 (1.7)	0 (0.0)	0.99
Total	60 (100.0)	34 (100.0)	-

**Table 3:** Antibiotic sensitivity and resistance of principal isolates to some common antibiotics

Organism	Antibiotic										
	Amoxycillin	Erythromycin	Cloxacillin	Co-trimoxazole	Co-trimoxazole	Chloramphenicol	Gentamicin	Ofloxacin	Ciprofloxacin	Ceftriazone	Cefuroxime
***Staph. aureus***											
***No. tested***	24	14	17	23	24	11	28	23	18	8	18
***% Resistance***	95.8	35.8	11.8	69.6	16.7	27.3	25.0	4.3	5.6	37.5	72.2
***%Sensitivity***	4.2	64.2	88.2	30.4	83.3	72.7	75.0	95.7	94.4	62.5	27.8
**Proteus spp.**											
***No. tested***	28	11	13	22	26	18	27	22	21	10	18
***% Resistance***	85.7	90.9	100.0	72.7	38.5	33.3	22.2	4.5	4.8	0.0	33.3
***%Sensitivity***	14.3	9.1	0.0	27.3	61.5	66.7	77.8	95.5	95.2	100.0	66.7
***Pseud. aeru.***											
***No. tested***	15	8	12	15	15	10	16	12	13	9	11
***% Resistance***	93.3	100.0	100.0	100.0	80.0	20.0	12.5	0.0	15.4	33.3	45.5
***%Sensitivity***	6.7	0.0	0.0	0.0	20.0	80.0	87.5	100.0	84.6	66.7	54.5
**Strep. Pneu.**											
***No. tested***	8	7	7	7	8	5	8	6	6	3	3
***% Resistance***	37.5	28.6	57.1	85.7	25.0	0.0	0.0	0.0	16.7	33.3	66.7
***%Sensitivity***	62.5	71.4	42.9	14.3	75.0	100.0	100.0	100.0	83.3	66.7	33.3

## Discussion

It was noted in this study that *Staphylococcus aureus*was the leading isolate among the acute cases of discharging otitis media, closely followed by Proteus species and *Pseudomonas aeruginosa*. This pattern seems to resemble observations by different researchers in Nigeria [5, 7] but does not mimic the trend in the developed world where non-typable *Haemophilus influenzae, Streptococcus pneumoniae* and *Moraxella catarrhalis* assume important predominant roles in acute otitis media [4, 11, 12]. Among the chronic cases of discharging otitis media, Proteus species was the leading causative isolate, followed closely by *Staphylococcus aureus*, and Pseudomonas aeruginosa. These three organisms seem to be the predominant ones in Europe, Middle East, [13] United States of America and within Nigeria [5, 14, 15]. *Streptococcus pneumoniae* was a less common isolate in this study; and this was likewise observed in Lagos [6]. The pattern of cultured isolates in chronically discharging ears is nearly similar to that observed by Tiedt and colleagues [16] in South Africa where the commonest isolates were *Proteus Mirabilis, Pseudomonas aeruginosa* and *Haemophilus influenzae*. The overall picture of bacteriology in this study also compares with the study carried out by Abera and Kibret in Ethiopia [17] and suggests that geographical location has a large part in the aetiology of discharging otitis media. Type of ear discharge did not seem to affect or influence the causative agent. Again, age of participants did not seem to have any bearing on the pattern of bacterial isolates. There is paucity of information in the literature regarding the possible role of age on the bacteriology of discharging otitis media. All the major isolates showed excellent *in vitro* sensitivity to the fluoroquinolone group of antibiotics (which in this study are ofloxacin and ciprofloxacin). This observation is same as that noted by Oni and fellow workers [5] in Ibadan. Although gentamycin and chloramphenicol had moderately good in vitro activity against *Staphylococcus aureus* and *Streptococcus pneumoniae* in this study, the research by Ako-Nai and colleagues [7] in Ile-Ife showed that these gram positive organisms were only weakly susceptible to gentamycin and chloramphenicol *in vitro*.

However, the number of isolates tested for sensitivity to gentamycin were five and three for *Staphylococcus aureus* and *Streptococcus pneumoniae respectively*, compared with twenty-eight and eight in this index study. This difference in number of isolates tested could well be a potential source of bias. On the other hand, in Ibadan (Nigeria) [5] and in Ethiopia [17], gentamycin was noted to have good antibacterial effectiveness against *Staphylococcus aureus*. The varying sensitivity of isolates to particular antibiotics found within a country and intracontinentally may well be a result of chronic and perhaps appropriateness of exposure of organisms to antibiotics. The fluoroquinolone group of antibiotics is a broad-spectrum class, which acts by inhibiting Deoxyribonucleic acid (DNA) gyrase. Its coverage includes the organisms most commonly associated with otitis media (*Streptococcus pneumoniae* regardless of susceptibility to penicillin, *Haemophilus influenzae*, *Moraxella catarrhalis* regardless of β-lactamase status, *Pseudomonas aeruginosa* and *Staphylococcus aureus*) [18]. Resistant strains are however emerging [19]. Systemic use of this group of antibiotics has been limited in children because of the observation that these antibiotics, when administered systemically, may have an adverse effect on the development of weight-bearing joints in juvenile animals [18, 19].

Ohyama and co-workers [20] in Japan demonstrated that 0.3% ofloxacin otic solution was efficacious without ototoxic effects in discharging otitis media of the chronic variety. Again, Dohar and fellow workers [18] showed that otic formulation of 0.3% ofloxacin in a dose of 0.25ml twice daily for 10 days eradicated cultured isolates in 96.3% of the participants tested without much adverse effects. Many researchers have documented good *in vitro* efficacy of gentamycin against *Pseudomonas aeruginosa*, Klebsiella species and Proteus species [5–7]. Indeed, Coker and colleagues [6] had observed that gentamycin appeared to be the most effective antibiotic against strains of *Pseudomonas aeruginosa*, Proteus and Klebsiella species and went on to recommend its topical application in chronic forms of middle ear discharge. Wariso and Ibe [21] in Port Harcourt and some other researchers in Ethiopia [17] also recommended it as the first drug of choice in treating chronic otitis media.

The results of this study suggest that there exists a high level of resistance of bacterial isolates to a number of commonly used antibiotics like cloxacillin, amoxicillin, erythromycin, ampiclox, co-trimoxazole, amoxicillin-clavulanate and even cefuroxime in *in vitro* testing. The increasing prevalence of multi-drug resistant bacteria is of epidemiological importance especially in attempts to control infection in the event of an epidemic caused by these agents. Such resistance may have arisen due to injudicious use of antibiotics especially as they are commonly used as 'over-the-counter'drugs with no qualified medical personnel's prescription. It is therefore being recommended that the Ministry of Health should restrict injudicious sale of antibiotics. They should only be sold on qualified health personnel's prescription to minimize their abuse. We also recommend that for acutely discharging ears, systemic Ofloxacin or ciprofloxacin could be used as the drug of choice until ear swab bacteriologic results are out. Gentamycin or ofloxacin or ciprofloxacin could serve as logical choice in chronically discharging ears until swab culture and sensitivity results are out.

## Conclusion

*Staphylococcal aureus* and Proteus species were the most common bacterial agents in acute and chronic otitis media respectively. Fluoroquinolones were found to be effective in their treatment.

### What is known about this topic

- Bacterial agents are known causes of both acute and chronic otitis media. Antibiotic therapy is key in the management of otitis media. In resource poor countries like Nigeria, empirical therapy is not uncommon, hence the need for implicating agents and susceptible antibiotics.

### What this study adds

- This study shows that the leading organisms in both acutely and chronically discharging otitis media among Nigerian children were *Staphylococcal aureus* and Proteus species, these organisms were more sensitive to fluoroquinolones group of antibiotics and should now be the first-line antibiotic management of otitis media among Nigerian children.
